# Cytogenetic Analysis Did Not Reveal Differentiated Sex Chromosomes in Ten Species of Boas and Pythons (Reptilia: Serpentes)

**DOI:** 10.3390/genes10110934

**Published:** 2019-11-15

**Authors:** Barbora Augstenová, Sofia Mazzoleni, Alexander Kostmann, Marie Altmanová, Daniel Frynta, Lukáš Kratochvíl, Michail Rovatsos

**Affiliations:** 1 Department of Ecology, Faculty of Science, Charles University, 12844 Prague, Czech Republic; augstenova.barbora@gmail.com (B.A.); sofia.mazzoleni@natur.cuni.cz (S.M.); alexkostmann@gmail.com (A.K.); altmanova.m@gmail.com (M.A.); lukas.kratochvil@natur.cuni.cz (L.K.); 2 Institute of Animal Physiology and Genetics, Czech Academy of Sciences, 27721 Liběchov, Czech Republic; 3 Department of Zoology, Faculty of Science, Charles University, 12844 Prague, Czech Republic; frynta@centrum.cz

**Keywords:** boa, comparative genomic hybridization, fluorescence in situ hybridization, evolution, karyotype, microsatellites, python, rDNA, sex chromosomes, sex determination, telomeres

## Abstract

Homologous and differentiated ZZ/ZW sex chromosomes (or derived multiple neo-sex chromosomes) were often described in caenophidian snakes, but sex chromosomes were unknown until recently in non-caenophidian snakes. Previous studies revealed that two species of boas (*Boa imperator*, *B. constrictor*) and one species of python (*Python bivittatus*) independently evolved XX/XY sex chromosomes. In addition, heteromorphic ZZ/ZW sex chromosomes were recently revealed in the Madagascar boa (*Acrantophis* sp. cf. *dumerili*) and putatively also in the blind snake *Myriopholis macrorhyncha*. Since the evolution of sex chromosomes in non-caenophidian snakes seems to be more complex than previously thought, we examined ten species of pythons and boas representing the families Boidae, Calabariidae, Candoiidae, Charinidae, Pythonidae, and Sanziniidae by conventional and molecular cytogenetic methods, aiming to reveal their sex chromosomes. Our results show that all examined species do not possess sex-specific differences in their genomes detectable by the applied cytogenetic methods, indicating the presence of poorly differentiated sex chromosomes or even the absence of sex chromosomes. Interestingly, fluorescence in situ hybridization with telomeric repeats revealed extensive distribution of interstitial telomeric repeats in eight species, which are likely a consequence of intra-chromosomal rearrangements.

## 1. Introduction

Snakes (Serpentes) are a clade of toxicoferan reptiles. With almost 3800 extant species, snakes represent about one third of all reptilian species [1]. Traditional taxonomy classifies snakes into three major lineages: Caenophidia, and the likely paraphyletic Scolecophidia and Henophidia [2,3,4,5]. Caenophidia is the most specious and diverse group, including more than 3100 species [1]. Scolecophidia is a group of approximately 400 species of blindsnakes with worm-like body shape and fossorial lifestyle. Henophidian snakes include about 200 species [1], recently divided into 21 families [3,6]. 

The diploid chromosomal number varies across snakes from 2n = 24 to 2n = 56 [7,8]. Phylogenetic reconstruction revealed that the ancestral snake karyotype consisted of 2n = 36 chromosomes with 16 macro- and 20 microchromosomes, which is the state commonly observed in the majority of the cytogenetically studied snake species [8]. 

Early pioneering cytogenetic studies revealed a ZZ/ZW sex determination system in several caenophidian snakes by conventional cytogenetic methods (e.g., karyotype reconstruction, C-banding) [7,8], which was later confirmed by molecular cytogenetic [9,10,11,12], bioinformatic [13], and molecular genetic [14] methods in all caenophidian families. The W chromosome of caenophidian snakes is highly degenerated and heterochromatic with accumulations of microsatellite motifs such as (TTAGGG)_n_ and (GATA)_n_, with variable topology and degree of accumulation among species [12]. On the contrary, sex-linked accumulation of repetitive sequences has not been reported so far in non-caenophidian snakes [11,15,16,17].

For a long time, it was assumed that non-caenophidian snakes exhibit ZZ/ZW sex determination with poorly differentiated sex chromosomes [13,18,19]. However, sporadic reports from non-caenophidian snakes, including the observations that (i) facultative parthenogenesis in pythons and boas leads to exclusively female progeny [20,21,22,23] and (ii) inheritance of a color mutation in the ball python, *Python regius* [24], indicate XX/XY sex determination. Recently, genomic studies confirmed the presence of XX/XY sex determination in three species of non-caenophidian snakes: the Burmese python *Python bivittatus,* the common boa *Boa constrictor,* and the northern boa *Boa imperator* [25]. Furthermore, the same study showed that the two boas and the python have non-homologous XX/XY sex chromosomes. However, a previous report of heteromorphic ZZ/ZW sex chromosomes in the Madagascar boa based on conventional cytogenetics [26] was recently confirmed by molecular cytogenetic methods in *Acrantophis* sp. cf. *dumerili* [27]. Another recent study showed that the scolecophidian long-nosed worm snake *Myriopholis macrorhyncha* may have heteromorphic ZZ/ZW sex chromosomes which are likely non-homologous to sex chromosomes of caenophidian snakes [28]. Therefore, in comparison to the long-term stability of the Z chromosome across all lineages of caenophidian snakes [14], the sex determination systems in non-caenophidian snakes are likely far less stable and more dynamic than was previously assumed.

In the present study, we examined the presence of differentiated sex chromosomes in ten species and one interspecific hybrid of snakes from six henophidian families: Boidae, Calabaridae, Candoiidae, Charinidae, Sanziniidae, and Pythonidae. We constructed karyograms and further explored their karyotypes by C-banding to reveal the distribution of constitutive heterochromatin. Furthermore, we applied comparative genomic hybridization (CGH) to identify sex-specific differences in the karyotypes and fluorescence in situ hybridization (FISH) with repetitive elements that often accumulate on the sex chromosomes of reptiles such as telomeric motifs, (GATA)_n_ microsatellite repeats, and rDNA loci [12,29,30,31].

## 2. Materials and Methods 

### 2.1. Studied Material, Chromosome Preparations, and Staining

We studied ten species belonging to six families of boas and pythons: *Chilabotrus angulifer*, *Eunectes notaeus* (Boidae), *Calabaria reinhardtii* (Calabariidae), *Candoia paulsoni* (Candoiidae), *Lichanura trivirgata* (Charinidae), *Morelia bredli*, *Morelia spilota* (Pythonidae), *Acrantophis dumerili*, *Acrantophis madagascariensis*, and *Sanzinia madagascariensis* (Sanziniidae) (Table 1). In addition, we studied an interspecific hybrid between *M. spilota* (father) and *M. bredli* (mother). Males and females were studied in all species with the exception of *M. bredli*, where we only had access to a female. 

From all studied specimens, we collected 1–2 ml of peripheral blood which was used for DNA isolation and whole blood cell cultures. For DNA isolation, we used the DNeasy Blood and Tissue Kit (Qiagen, Valencia, CA, USA) following the manufacturer’s protocol. Mitotic chromosome suspensions were prepared from whole blood cell cultures according to the protocol described in Mazzoleni et al. [32].

Chromosome preparations were stained by Giemsa for karyogram reconstruction. For visualization of constitutive heterochromatin, we applied C-banding following the protocol of Sumner [33] with slight modifications. Briefly, slides were treated in 0.2 N HCl for 30 minutes at room temperature, in 5% Ba(OH)_2_ for 5 minutes at 45 °C, in 2× saline-sodium citrate (SSC) buffer for 1 h at 60 °C, washed with distilled water, and air-dried. The slides were stained by Fluoroshield with DAPI (4′,6-diamidino-2-phenylindole; Sigma-Aldrich, St. Louis, MO, USA). The results of C-banding from both sexes of *Acrantophis dumerili, Acrantophis madagascariensis,* and *Sanzinia madagascariensis* were published in the previous paper [27], and we did not repeat them here.

### 2.2. Fluorescence In Situ Hybridization with Telomeric Probe

We prepared the telomeric probe (TTAGGG)_n_ by polymerase chain reaction (PCR) without a DNA template using the primers (TTAGGG)_5_ and (CCCTAA)_5_ according to the protocol of Ijdo et al. [34]. Primers were synthesized by Macrogen (Seoul, Korea). The probe was precipitated using salmon sperm, sodium acetate (3M), and ethanol. The precipitated probe was dissolved in hybridization buffer (50% formamide in 2× SSC). 

Slides with chromosome spreads were treated according to our standard protocols described in Rovatsos et al. [35]. Briefly, the slides were washed in 2× SSC, treated with RNAse for 1 h at 37 °C, and washed in 2× SSC. The slides were subsequently treated with pepsin for 10 min at 37 °C, washed in phosphate buffered saline (PBS), incubated for 10 minutes in 1% solution of formaldehyde in 2× SSC, washed again in PBS, and then dehydrated using ethanol series. In the next step, the chromosomes were denatured in 70% formamide in 2× SSC at 75 °C for 4 min, washed in 2× SSC, and again dehydrated through ethanol series. Subsequently, we added 11 μL of probe to each slide and incubated the slides overnight at 37 °C. The following day, we washed the slides in 2× SSC and then in 50% formamide in 2× SSC at 40 °C (three times for 5 minutes). Slides were subsequently washed in 2× SSC and 4× SSC / 0.05% Tween20 (Sigma). Subsequently, the slides were incubated with 4× SSC / 5% blocking reagent (Roche, Basel, Switzerland) at 37 °C for 45 min. In the next step, we added 4× SSC / 5% blocking reagent containing avidin-FITC (Vector Laboratories, Burlingame, CA, USA). We amplified the fluorescence signal with a modified avidin-FITC/biotinylated anti-avidin system (Vector Laboratories, Burlingame, CA, USA). The slides were dehydrated by ethanol series and stained by Fluoroshield with DAPI.

### 2.3. Fluorescence In Situ Hybridization with rDNA Probe

The probe for FISH with rDNA loci was prepared from a plasmid (pDmr.a 51#1) with an 11.5-kb insert encoding the 18S and 28S ribosomal units of *Drosophila melanogaster* [36] and was labelled with dUTP-biotin using nick translation (Abbott Molecular, Lake Bluff, IL, USA). The rDNA probe was hybridized to chromosome preparations and detected according to the above described protocol.

### 2.4. Fluorescence In Situ Hybridization with (GATA)_n_ Probe

The probe for the (GATA)_n_ microsatellite motif was synthesized and biotin-labelled by Macrogen (Korea). The probe was resuspended in hybridization buffer (50% formamide, 10% sodium dodecyl sulphate (SDS), 10% dextran sulphate, Denhard’s buffer, 2× SSC, pH 7). We followed the same protocol used for FISH with the telomeric probe, except that the post-hybridization washes were performed in 0.4% Nonidet P-40 in 2× SSC (Sigma-Aldrich) at 40 °C for 2 min and then in 0.1% Nonidet P-40 in 0.4× SSC at room temperature for 30s, instead of 50% formamide in 2× SSC.

### 2.5. Comparative Genomic Hybridization

The protocol followed Rovatsos et al. [37]. Briefly, male and female genomic DNA were labelled with biotin-deoxyuridine triphosphate (dUTP) and digoxigenin-dUTP, respectively, using a Nick Translation Kit (Abbott Laboratories, Lake Bluff, IL, USA). 1 μg of male and 1 μg of female labelled genomic DNA were co-precipitated overnight with 5 μL of salmon sperm DNA (10 mg/mL, Sigma), 10 μL of 3M sodium acetate, and 2.5× volume of ethanol. After precipitation, the dry pellets were resuspended in 22 μL of hybridization buffer (50% formamide, 2× SSC, 10% SDS, 10% dextran sulfate, 1× Denhardt’s buffer, pH 7), denatured at 75 °C for 10 min, and subsequently chilled on ice for 10 min prior to hybridization. In parallel, the slides with chromosome preparations were treated with RNase and pepsin, fixed with 4% formaldehyde, dehydrated through a 70%, 85%, and 100% ethanol series, denatured in 70% formamide / 2× SSC at 75 °C for 3 min, and dehydrated again. 11 μL of the probe (concentration approximately 300–700 ng of labeled DNA) was applied to the slide per drop of chromosomal suspension and incubated at 37 °C for 48 h. Post-hybridization washes were performed in 50% formamide / 2× SSC at 42 °C and in 2× SSC. Each slide was incubated with 100 μL of 4× SSC / 5% blocking reagent (Roche) at 37 °C for 30 min and then with 100 μL of detection solution 4× SSC / 5% blocking reagent including 2 μL of avidin-FITC (Vector Laboratories, Burlingame, CA, USA) and 10 μL of anti-digoxigenin-rhodamine (Roche, Basel, Switzerland) at 37 °C for 30 min. The slides were washed in 4× SSC / 0.05% Tween 20, dehydrated through an ethanol series, and air dried. Finally, the slides were mounted with Fluoroshield antifade medium containing DAPI (Sigma-Aldrich, St. Louis, MO, USA). The results of CGH in *S. madagascariensis* and *Acrantophis* sp. cf. *dumerili* (closely related to *A. dumerili* and *A. madagascariensis*) were published by us in the previous paper [27]; therefore, we do not present the results of CGH for sanziniid snakes here. We were not able to perform CGH in *Morelia bredli*, where we had access to only a single sex; however, CGH was performed in the interspecific hybrids, whose mother was *M. bredli* and father *M. spilota*.

### 2.6. Microscopy and Image Analyses

Zeiss Axio Imager Z2, equipped with automatic Metafer-MSearch scanning platform (MetaSystems) and a CoolCube 1 b/w digital camera (MetaSystems, Altlussheim, Germany) was used to capture Giemsa-stained metaphases. Karyograms were prepared with software Ikaros (MetaSystems). A Provis AX70 (Olympus, Tokyo, Japan) fluorescence microscope equipped with a DP30BW digital camera (Olympus) was used to capture images from the FISH experiments, which were subsequently processed in DP MANAGER imaging software (Olympus).

## 3. Results

**Figure 1:**
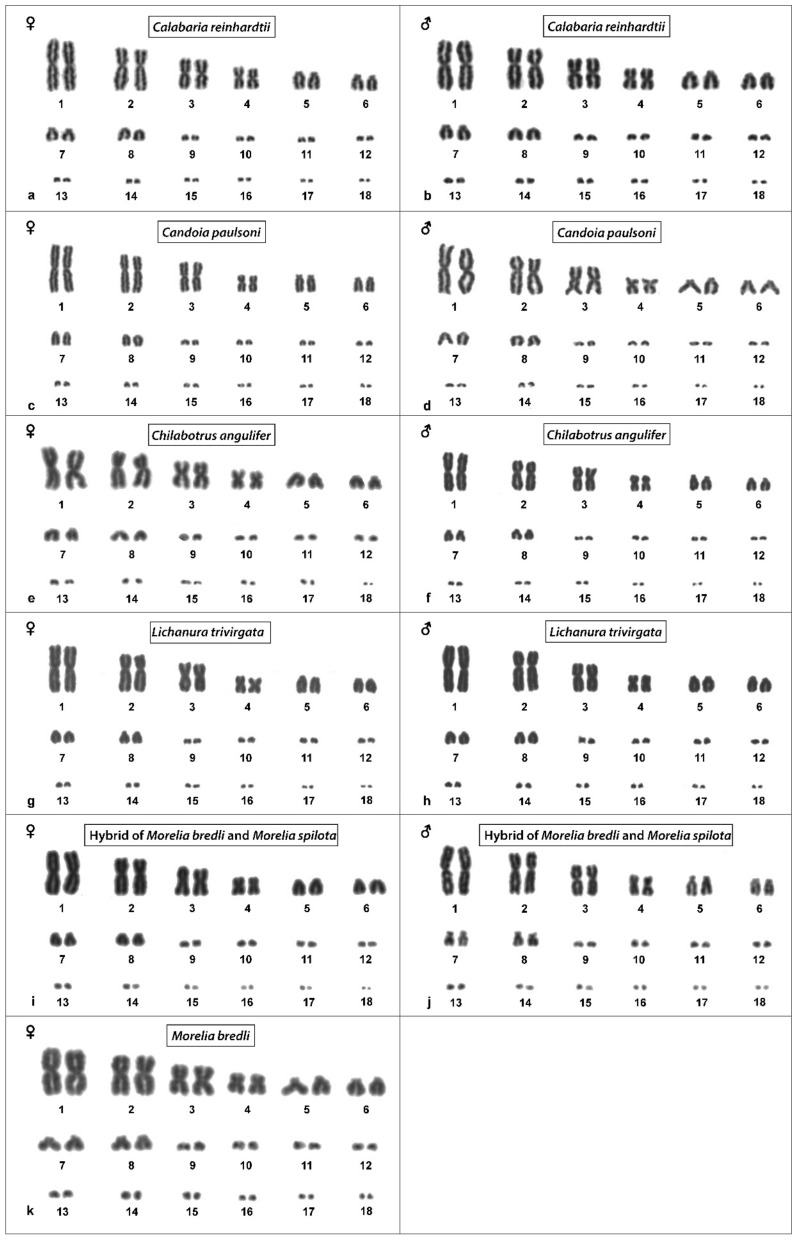
Giemsa-stained karyograms of *Calabaria reinhardtii* (**a**,**b**), *Candoia paulsoni* (**c**,**d**), *Chilabothrus angulifer* (**e**,**f**), *Lichanura trivirgata* (**g**,**h**), *Morelia bredli × M. spilota* hybrid (**i**,**j**) and *Morelia bredli* (**k**).

**Figure 2:**
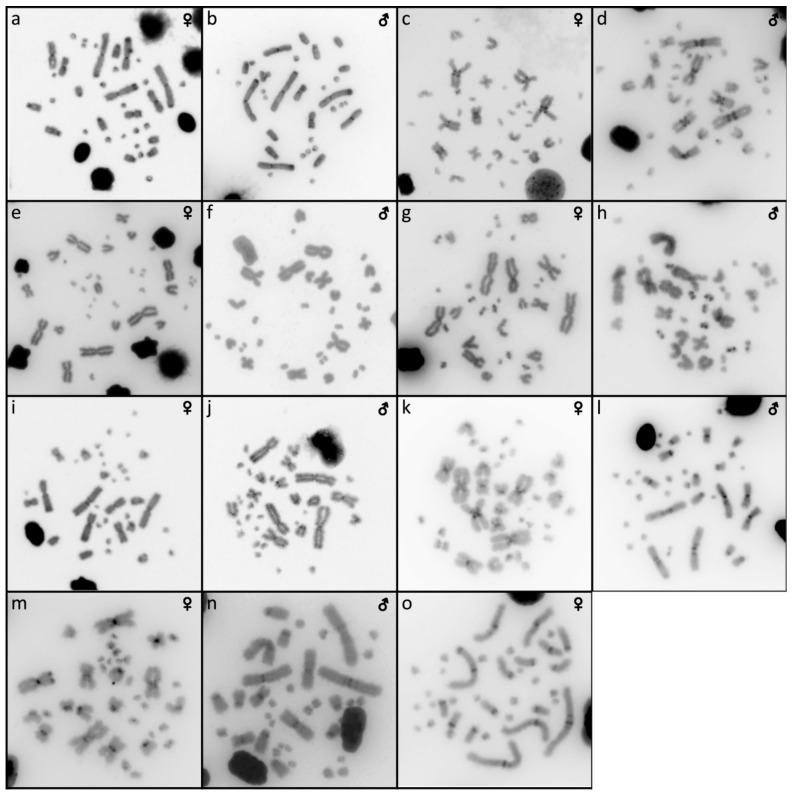
Distribution of heterochromatic blocks uncovered by C-banding in metaphases of *Calabaria reinhardtii* (**a**,**b**)*, Candoia paulsoni* (**c**,**d**)*, Chilabothrus angulifer* (**e**,**f**)*, Eunectes notaeus* (**g**,**h**)*, Lichanura trivirgata* (**i**,**j**), *Morelia spilota* (**k**,**l**)*, Morelia bredli* × *M. spilota* hybrid (**m**,**n**) and *Morelia bredli* (**o**).

**Figure 3:**
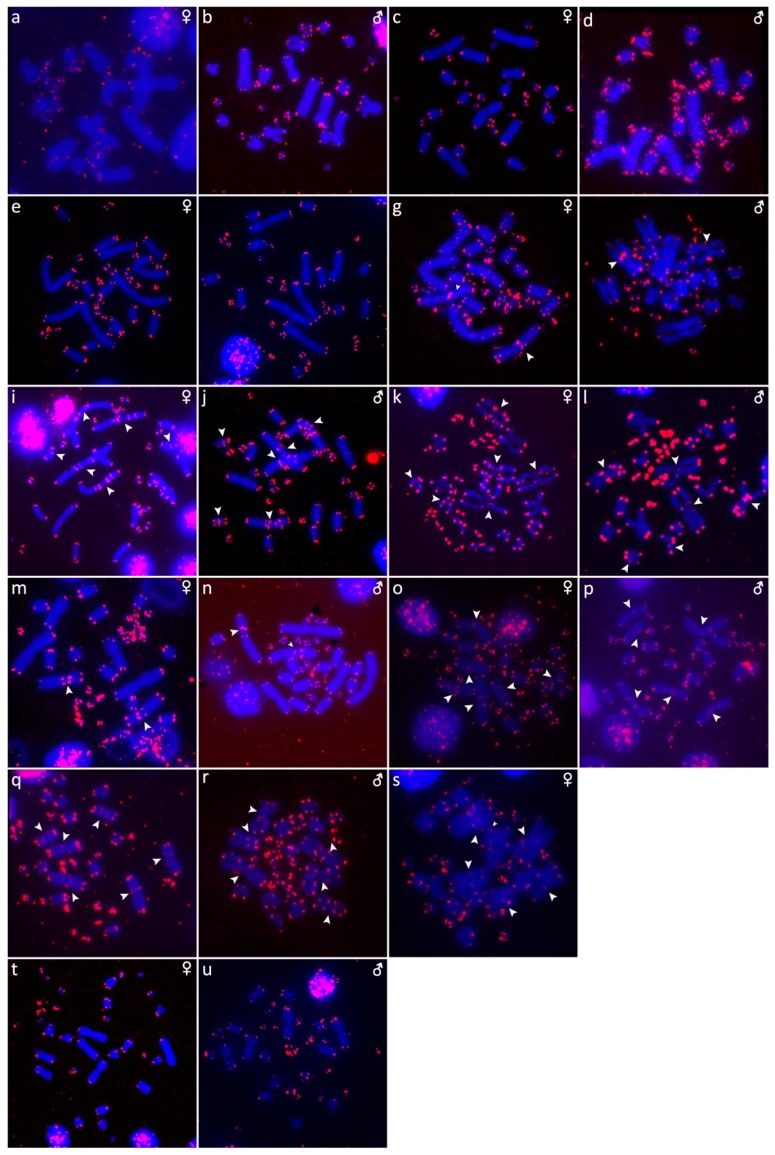
Distribution of (TTAGGG)_n_ motifs in metaphases of *Acrantophis dumerili* (**a**,**b**), *Acrantophis madagascariensis* (**c**,**d**), *Calabaria reinhardtii* (**e**,**f**), *Candoia paulsoni* (**g**,**h**), *Chilabothrus angulifer* (**i**,**j**), *Eunectes notaeus* (**k**,**l**), *Lichanura trivirgata* (**m**,**n**), *Morelia spilota* (**o**,**p**), *Morelia bredli × M. spilota* hybrid (**q**,**r**), *Morelia bredli* (**s**), and *Sanzinia madagascariensis* (**t**,**u**). Chromosomes with interstitial telomeric repeats are shown by arrows.

**Figure 4:**
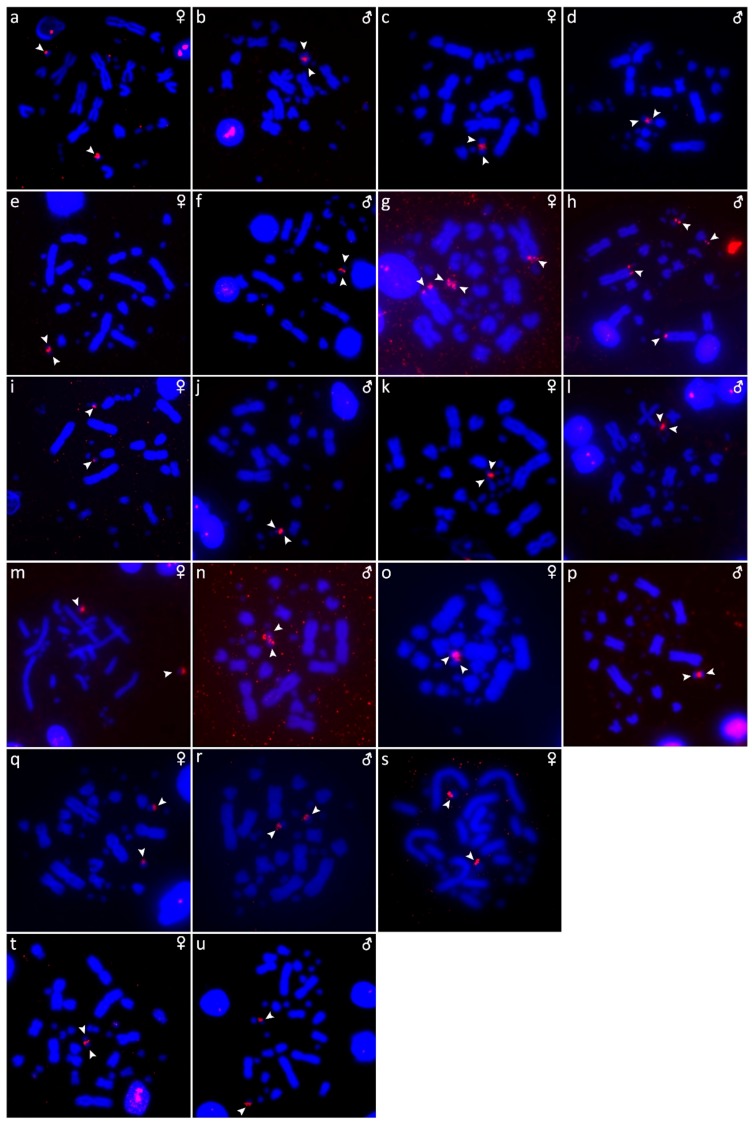
Topology of rDNA loci in metaphases of *Acrantophis dumerili* (**a**,**b**), *Acrantophis madagascariensis* (**c**,**d**), *Calabaria reinhardtii* (**e**,**f**), *Candoia paulsoni* (**g**,**h**), *Chilabothrus angulifer* (**i**,**j**), *Eunectes notaeus* (**k**,**l**), *Lichanura trivirgata* (**m**,**n**), *Morelia spilota* (**o**,**p**), *Morelia bredli × M. spilota* hybrid (**q**,**r**), *Morelia bredli* (**s**), and *Sanzinia madagascariensis* (**t**,**u**). Chromosomes with detected signal are shown by arrows.

The results are presented in the figures according to the technique used in the order: Giemsa-stained karyograms of previously cytogenetically unstudied species (Figure 1), C-banding (Figure 2), distribution of (TTAGGG)_n_ motifs (Figure 3), rDNA accumulations (Figure 4) and (GATA)_8_ motifs, and CGH (Figure 6).

### 3.1. Acrantophis Dumerili

**Figure 5:**
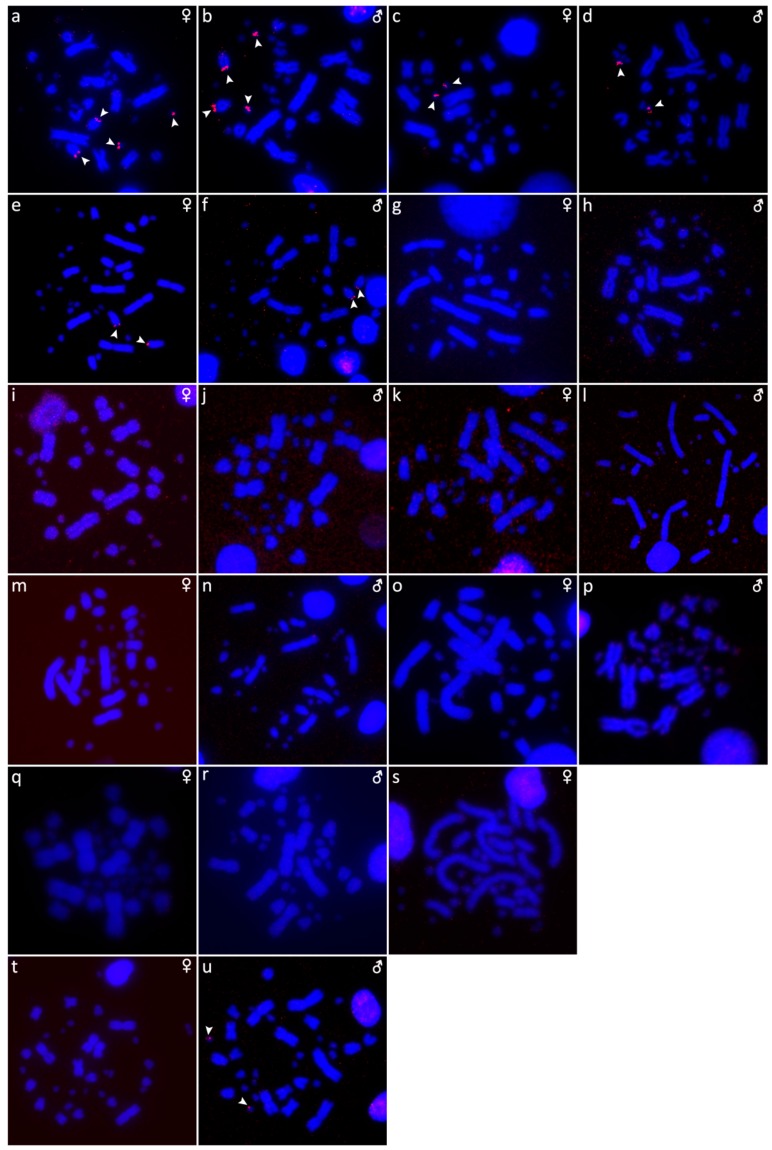
Distribution of (GATA)_8_ motifs in metaphases of *Acrantophis dumerili* (**a**,**b**), *Acrantophis madagascariensis* (**c**,**d**), *Calabaria reinhardtii* (**e**,**f**), *Candoia paulsoni* (**g**,**h**), *Chilabothrus angulifer* (**i**,**j**), *Eunectes notaeus* (**k**,**l**), *Lichanura trivirgata* (**m**,**n**), *Morelia spilota* (**o**,**p**), *Morelia bredli × M. spilota* hybrid (**q**,**r**), *Morelia bredli* (**s**), and *Sanzinia madagascariensis* (**t**,**u**). Chromosomes with detected signal are shown by arrows.

The karyotype is composed of 2n = 34 chromosomes (16 macro- and 18 microchromosomes), as was previously described in Augstenová et al. [27], where the results of C-banding were presented. The telomeric sequences were detected only in terminal positions of the chromosomes (Figure 3a,b). FISH with rDNA probe revealed signals on a pair of microchromosomes (Figure 4a,b). FISH with (GATA)_8_ motif revealed signals on the 5th pair and a pair of microchromosomes (Figure 5a,b).

### 3.2. Acrantophis Madagascariensis

The karyotype consists of 2n = 34 chromosomes (16 macro- and 18 microchromosomes) as was previously reported in Augstenová et al. [27], where the results of C-banding can be found. The telomeric sequences were detected only at terminal positions of the chromosomes (Figure 3c,d). FISH with rDNA probe revealed signals on a pair of microchromosomes (Figure 4c,d). FISH with (GATA)_8_ motif revealed signals on a pair of microchromosomes (Figure 5c,d).

### 3.3. Calabaria Reinhardtii

**Figure 6:**
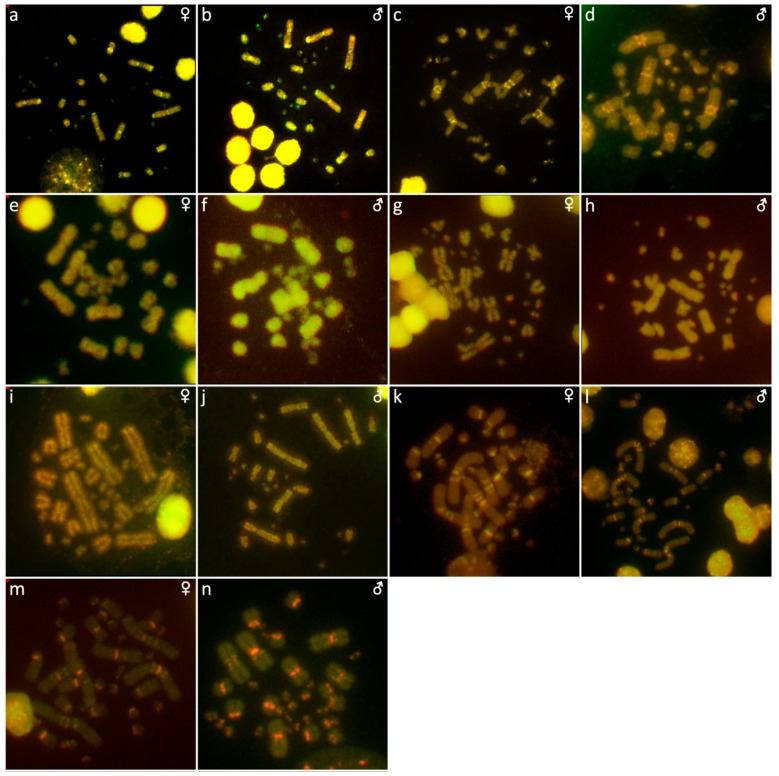
Comparative genomic hybridization in metaphases of *Calabaria reinhardtii* (**a**,**b**), *Candoia paulsoni* (**c**,**d**), *Chilabothrus angulifer* (**e**,**f**), *Eunectes notaeus* (**g**,**h**), *Lichanura trivirgata* (**i**,**j**), *Morelia spilota* (**k**,**l**), and *Morelia bredli × M. spilota* hybrid (**m**,**n**).

The karyotype consists of 2n = 36 chromosomes (16 macro- and 20 microchromosomes). The pairs 1–4 consist of bi-armed chromosomes (metacentric or submetacentric), while the pairs 5–8 consist of acrocentric chromosomes (Figure 1a,b). C-banding revealed heterochromatin mainly in (peri-) centromeric regions (Figure 2a,b). The telomeric sequences were detected only at terminal positions of the chromosomes (Figure 3e,f). FISH with rDNA probe revealed signals on a pair of microchromosomes (Figure 4e,f). FISH with (GATA)_8_ revealed signals on the 5th pair (Figure 5e,f). We did not detect any sex-specific differences at sequence level by CGH (Figure 6a,b).

### 3.4. Candoia Paulsoni

The karyotype consists of 2n = 36 chromosomes (16 macro- and 20 microchromosomes). The pairs 1–4 are bi-armed chromosomes, while the pairs 5–8 are acrocentric (Figure 1c,d). C-banding revealed heterochromatin in pericentromeric regions of macrochromosomes (Figure 2c,d). The telomeric sequences were detected at terminal positions of the chromosomes. In addition, interstitial telomeric repeats (ITRs) were detected in the 2nd pair (Figure 3g,h). Accumulation of rDNA motifs was detected at terminal positions of the first pair and in a pair of microchromosomes (Figure 4g,h). FISH with (GATA)_8_ did not reveal a signal on any chromosomes (Figure 5g,h). We did not detect any sex-specific differences at sequence level by CGH (Figure 6c,d).

### 3.5. Chilabothrus Angulifer

The karyotype is composed of 2n = 36 chromosomes (16 macro- and 20 microchromosomes). The pairs 1–4 consist of bi-armed chromosomes, while the pairs 5–8 consist of acrocentric chromosomes (Figure 1e,f). C-banding did not reveal any accumulations of heterochromatin (Figure 2e,f). The telomeric sequences were detected at terminal positions of the chromosomes. In addition, ITRs were detected in the 1st and 2nd chromosome pair (Figure 3i,j). FISH with rDNA probe revealed signals on a pair of microchromosomes (Figure 4i,j). FISH with (GATA)_8_ motif did not reveal a signal on any chromosome (Figure 5i,j). We did not detect any sex-specific differences at sequence level by CGH (Figure 6e,f).

### 3.6. Eunectes Notaeus

The karyotype is composed of 2n = 36 chromosomes (16 macro- and 20 microchromosomes) as was previously described [17]. C-banding revealed a strong accumulation of heterochromatin in pericentromeric regions (Figure 2g,h). The telomeric sequences were detected in terminal positions of the chromosomes. FISH with telomeric probe uncovered ITRs on the 1^st^, 2nd, and 6th chromosomal pairs (Figure 3k,l). rDNA was accumulated in one pair of microchromosomes (Figure 4k,l). FISH with probe specific for (GATA)_8_ motif did not reveal a signal on any chromosomes (Figure 5k,l). We did not detect any sex-specific differences at sequence level by CGH (Figure 6g,h).

### 3.7. Lichanura Trivirgata

The karyotype is composed of 2n = 36 chromosomes (16 macro- and 20 microchromosomes). The pairs 1–4 consist of bi-armed chromosomes, while the pairs 5–8 consist of acrocentric chromosomes (Figure 1g,h). C-banding revealed heterochromatin mainly in centromeric regions (Figure 2i,j). The telomeric sequences were detected in terminal positions of the chromosomes. In addition, ITRs were detected in the 2nd chromosome pair (Figure 3m,n). FISH with rDNA probe revealed signals on a pair of microchromosomes (Figure 4m,n). FISH with probe specific for (GATA)_8_ motif did not reveal a signal on any chromosomes (Figure 5m,n). We did not detect any sex-specific differences at sequence level by CGH (Figure 6i,j).

### 3.8. Morelia Bredli

The karyotype consists of 36 chromosomes (16 macro- and 20 microchromosomes). The pairs 1–4 consist of bi-armed chromosomes, while the pairs 5–8 consist of acrocentric chromosomes (Figure 1k). C-banding revealed distribution of heterochromatin in pericentromeric regions (Figure 2o). The telomeric sequences were detected in terminal positions of the chromosomes. In addition, we detected ITRs in the 1^st^, 2nd, and 3rd pairs (Figure 3s). rDNA loci were accumulated on a pair of microchromosomes (Figure 4s). FISH with (GATA)_8_ repetitive elements did not detect any sex-specific accumulation (Figure 5s). A male of this species was not available to us at the time of the study, therefore we did not apply the CGH method.

### 3.9. Morelia Spilota

The karyotype consists of 36 chromosomes with 16 macro- and 20 microchromosomes, as was previously described [7,38]. C-banding uncovered heterochromatin regions around centromeres (Figure 2k,l). The telomeric sequences were detected in terminal positions of the chromosomes. In addition, we detected ITRs on the 1^st^, 2nd, and 3rd pairs (Figure 3o,p). FISH with rDNA probe uncovered a signal on one pair of microchromosomes (Figure 4o,p). FISH with (GATA)_8_ probe did not reveal any strong accumulation (Figure 5o,p). CGH did not revealed any sex-specific differences (Figure 6k,l).

### 3.10. Morelia Bredli × Morelia Spilota Hybrid

The karyotype of the interspecific hybrid between *Morelia bredli* and *Morelia spilota* is composed of 36 chromosomes with 16 macro- and 20 microchromosomes (Figure 1i,j). Heterochromatin regions are mostly restricted to pericentromeric regions as in the maternal species (Figure 2m,n). The telomeric sequences were detected in terminal positions of the chromosomes. In addition, we detected ITRs in the 1^st^, 2nd, and 3rd pair (Figure 3q,r). FISH with probe for rDNA loci revealed a signal on one pair of microchromosomes (Figure 4q,r). FISH with (GATA)_8_ probe did not reveal any sex-specific accumulation (Figure 5q,r). CGH of maternal and paternal species detected no species-specific differences (Figure 6m,n).

### 3.11. Sanzinia Madagascariensis

The karyotype consists of 34 chromosomes with 18 macro- and 16 microchromosomes, as previously described [26,27]. The results of C-banding and CGH can be found in [27]. The telomeric sequences were detected in terminal positions of the chromosomes (Figure 3t,u). FISH with the rDNA probe uncovered a signal on one pair of microchromosomes (Figure 4t,u). Accumulation of (GATA)_8_ microsatellite was detected in one pair of microchromosomes in the male, but not in the female (Figure 5t,u).

## 4. Discussion

According to our knowledge, we present the first description of karyotypes of five species, namely *Calabaria reinhardtii* (Calabariidae), *Candoia paulsoni* (Candoiidae), *Chilabothrus angulifer* (Boidae), *Lichanura trivirgata* (Charinidae), and *Morelia bredli* (Pythonidae) (Figure 1). 

Cytogenetic analysis revealed variability among species in the topology of the telomeric repeats, rDNA loci, and (GATA)_n_ motifs. 

**Figure 7:**
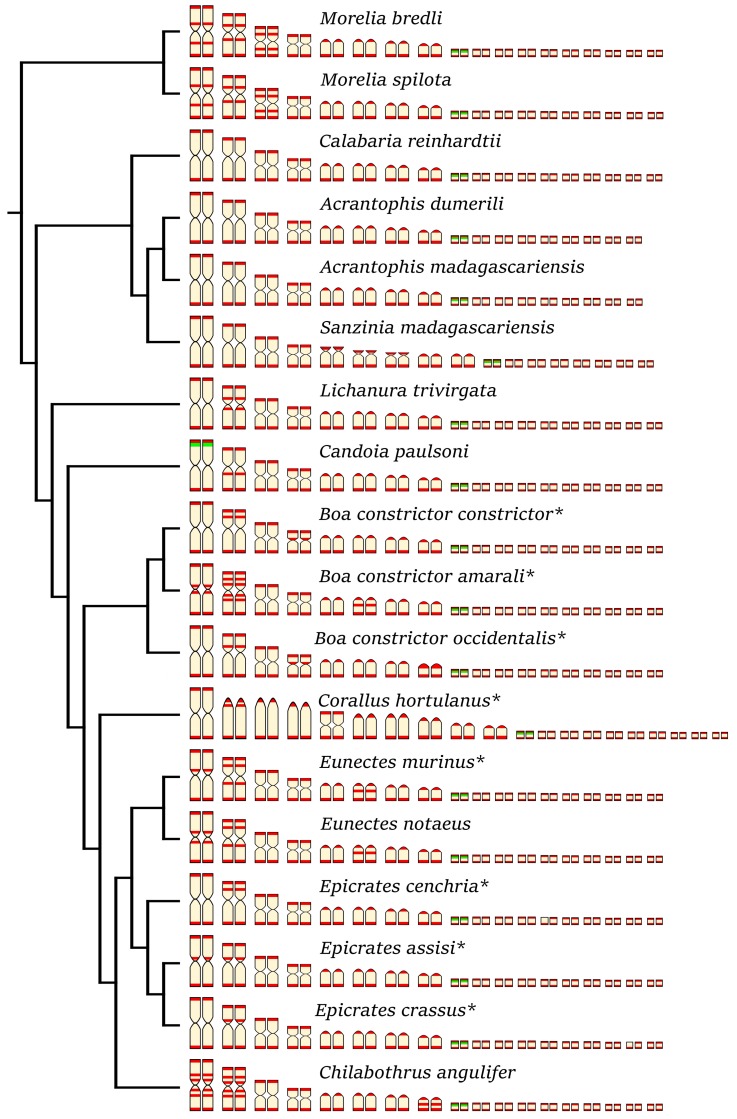
Idiogram showing the comparative topology of ITRs (in red) and rDNA loci (in green) on the chromosomes of 18 species of boas and pythons. The original data were extracted from Viana et al. [17] for species marked with asterisk (*). The phylogenetic relationships follow Reynolds et al. [39].

We detected interstitial telomeric repeats in six of the ten studied species (Figure 3 and Figure 7). Notably, in contrast to a previous study that reported telomeric repeats only in terminal positions [17], we detected ITRs in three individuals of *Eunectes notaeus*. Boas and pythons have karyotypes with similar chromosome morphology (Figure 7). Therefore, we assume that interchromosomal rearrangements occur rarely in these groups, and that the ITRs in the six studied species emerged via intrachromosomal rearrangements, such as inversions, or retrotransposon activity. The extensive polymorphism in the topology of ITRs does not follow a notable phylogenetic pattern (Figure 7). 

rDNA loci seem to accumulate on one pair of microchromosomes in all studied species (Figure 4), with the exception of *C. paulsoni*, which has an additional accumulation of rDNA on the 1st pair (Figure 4g,h). 

The (GATA)_n_ motif is accumulated in the genomes of boas and pythons only rarely (Figure 5). This accumulation was observed on the 5th chromosome pair in *Calabaria reinhardtii,* the 5th pair (Figure 5e,f) and a single pair of microchromosomes in *Acrantophis dumerili* (Figure 5a,b), and a pair of microchromosomes in *A. madagascariensis* (Figure 5c,d). The (GATA)_n_ motif was observed on one pair of microchromosomes in male metaphases of *Sanzinia madagascariensis*, but such accumulation was not detected in female metaphases (Figure 5t,u). It seems that this pattern reflects individual variability in *S. madagascariensis*.

We attempted to identify sex chromosomes in ten species of boas and pythons by both conventional and molecular cytogenetic methods. Sex-specific differences were not detected in the C-banded metaphases, indicating that sex chromosomes in the studied species are not heterochromatinized (Figure 2). Previous studies revealed an accumulation of telomeric-like sequences on the W sex chromosome in the lacertid lizard *Lacerta agilis* [40] and in the gecko *Underwoodisaurus milii* [41] as well as in several caenophidian snakes, including the dragon snake *Xenodermus javanicus* (Xenodermatidae) [9] and the masked water snake *Homalopsis buccata* (Homalopsidae) [12]. Some reptilian species, such as the red-bellied short-necked turtle *Emydura subglobosa* [42], the Chinese softshell turtle *Pelodiscus sinensis* [43], and the spiny softshell turtle *Apalone spinifera* [44], have accumulations of rDNA loci on sex chromosomes. Furthermore, the *Bkm* repeat, is composed of repetitions of (GATA)_n_ and (GACA)_n_ motifs [15,16], which commonly amplify in vertebrate sex chromosomes, including those of the vast majority of studied caenophidian snakes [12,30,31,45]. Our results did not reveal sex-specific accumulations of the telomeric repeats, rDNA loci, nor (GATA)_n_ motifs in the ten studied species of boas and pythons, with a single exception being the dubious male-specific accumulation of the (GATA)_n_ motif in *Sanzinia madagascariensis*. Comparative genomic hybridization (CGH) did not reveal sex-specific differences at the sequence level (up to the detection efficiency of in situ hybridization methodology) in all six tested species (Figure 6).

The differentiation process of sex chromosomes, including heterochromatinization, amplification of repetitive elements, and loss of functional genes in the heterogametic sex [46], seems to act as an “evolutionary trap”, prohibiting sex chromosome turnover and thus stabilizing the sex determination system [47]. Snakes seem to support this hypothesis. The ZZ/ZW sex determination system of caenophidian snakes with highly differentiated sex chromosomes seems to have been stable for more than 60 million years [9]. In contrast, henophidian and scolecophidian snakes have poorly differentiated (if any) sex chromosomes [17,28,48,49] and demonstrate variability in sex determination systems [25,27,28].

The evolution of sex chromosomes and sex determination systems in henophidian and scolecophidian snakes should be further explored by genome-wide next generation sequencing approaches, such as restriction site associated DNA sequencing (RAD-seq) [25,50,51,52], which will allow the fine-tuned identification of sex-specific regions of the genome. Facultative parthenogenetic reproduction results in all-female homozygous progeny in *Epicrates maurus* [20], *Epicrates cenchria cenchria* [22], *Malayopython reticulatus,* and *Python regius* [23], which indicates that male heterogamety may be present in these species. Under male heterogamety, the mother with XX sex chromosome constitution could produce only female offspring from facultative asexual reproduction. In contrast, under female heterogamety, the ZW mother should produce offspring of both sexes, or exclusively ZZ males, depending on the degree of W chromosome degeneration and the viability of the WW individuals [53]. Despite genotypic sex determination being exclusively revealed in all studied snakes and commonly expected in this lineage [54,55], we cannot exclude the possibility that some non-caenophidian snakes might possess environmental sex determination. Temperature and hydric conditions can induce sex-differential mortality in snake embryos [56,57,58] and can alter neonate morphology, physiology, and behavior (reviewed in [55]). The sex determination mode is usually determined either by cytogenetic examination (restricted to species with differentiated sex chromosomes) or by incubating eggs using a range of temperatures in laboratory conditions and examining the variation in hatchlings’ sex ratio [55]. However, most examined henophidian and scolecophidian snakes lack differentiated sex chromosomes and are not convenient for manipulative breeding experiments, as many of them are viviparous, lay small clutches, or rarely breed in captivity. We suggest that additional research is needed for uncovering the sex-determination mode in non-caenophidian snakes.

## Figures and Tables

**Table 1:** Summary of studied species and number of examined individuals.

Family	Species	♀	♂
Boidae	*Chilabothrus angulifer*	2	1
	*Eunectes notaeus*	1	2
Calabariidae	*Calabaria reinhardtii*	1	2
Candoiidae	*Candoia paulsoni*	1	1
Charinidae	*Lichanura trivirgata*	1	1
Sanziniidae	*Acrantophis dumerili*	7	3
	*Acrantophis madagascariensis*	2	1
	*Sanzinia madagascariensis*	1	1
Pythonidae	*Morelia bredli*	1	0
	*Morelia spilota*	3	4
	*Morelia bredli × Morelia spilota* hybrid	3	1
